# Diversity in Stakeholder Groups in Generative Co-design for Digital Health: Assembly Procedure and Preliminary Assessment

**DOI:** 10.2196/38350

**Published:** 2023-02-14

**Authors:** Pieter Vandekerckhove, Job Timmermans, Antoinette de Bont, Marleen de Mul

**Affiliations:** 1 Erasmus School of Health Policy & Management Erasmus University Rotterdam Rotterdam Netherlands; 2 Department of Military Management Studies Netherlands Defence Academy Breda Netherlands; 3 Tilburg School of Social & Behavioral Sciences Tilburg University Tilburg Netherlands

**Keywords:** collaborative design, design methodology, stakeholder involvement, participatory design, digital health

## Abstract

**Background:**

Diverse knowledge and ways of thinking are claimed to be important when involving stakeholders such as patients, care professionals, and care managers in a generative co-design (GCD) process. However, this claim is rather general and has not been operationalized; therefore, the influence of various stakeholders on the GCD process has not been empirically tested.

**Objective:**

In this study, we aimed to take the first step in assessing stakeholder diversity by formulating a procedure to assemble a group of diverse stakeholders and test its influence in a GCD process.

**Methods:**

To test the procedure and assess its influence on the GCD process, a case was selected involving a foundation that planned to develop a serious game to help people with cancer return to work. The procedure for assembling a stakeholder group involves snowball sampling and individual interviews, leading to the formation of 2 groups of stakeholders. Thirteen people were identified through snowball sampling, and they were briefly interviewed to assess their knowledge, inference experience, and communication skills. Two diverse stakeholder groups were formed, with one more potent than the other. The influence of both stakeholder groups on the GCD process was qualitatively assessed by comparing the knowledge output and related knowledge processing in 2 identical GCD workshops.

**Results:**

Our hypothesis on diverse stakeholders was confirmed, although it also appeared that merely assessing the professional background of stakeholders was not sufficient to reach the full potential of the GCD process. The more potently diverse group had a stronger influence on knowledge output and knowledge processing, resulting in a more comprehensive problem definition and more precisely described solutions. In the less potently diverse group, none of the stakeholders had experience with abduction-2 inferencing, and this did not emerge in the GCD process, suggesting that at least one stakeholder should have previous abduction-2 experience.

**Conclusions:**

A procedure to assemble a stakeholder group with specific criteria to assess the diversity of knowledge, ways of thinking, and communication can improve the potential of the GCD process and the resulting digital health.

## Introduction

### Background

Stakeholders such as patients, care professionals, and care managers are considered to play an important role in designing and creating digital health [1-4]. A widely used form of co-design that can involve a group of people to develop a digital health product is generative co-design (GCD) [5,6]. GCD is characterized by a collective creative process whereby knowledge is shared by stakeholders to develop a product or service, such as digital health [7-12]. In a GCD process, stakeholders are more actively involved in the creative design process than in a more classical design process [10].

A wide variety of people who do not necessarily have a design background, such as patients, care professionals, and health policy makers, can be GCD stakeholders in a digital health project. For instance, content experts such as patients (often referred to as “users”) may improve the uptake of the output, as their needs regarding user guidance, specific reminders, and personal tracking will likely be better addressed [13]. Health policy experts may also contribute to digital health development. For instance, it has been suggested that their involvement during the COVID-19 pandemic has led to improved alignment between payers and care professionals, which may have contributed to the rapid uptake of digital health [14,15].

There are both theoretical and practical issues when involving different stakeholders in GCD. From a theoretical standpoint, GCD scholars hypothesize that the more the diverse stakeholders involve in a group in terms of diverse knowledge and ways of thinking, the better the GCD process [10]. However, this claim is not clearly explicated, which may be due to the conceptual challenges present, such as the lack of consensus on the definition of “stakeholder” and “involvement” [16]. For instance, how one defines involvement depends on how one views stakeholder representation, the time involved in the project, and whether the scope focuses on the project or a wider cultural change [16-18]. In addition, GCD is part of a larger research field known as participatory design (PD) [10]. In PD, specific values are upheld, including democracy, equalized power relations, mutual learning, and situation-based actions [16,19]. However, these values are not currently applied explicitly in the GCD stakeholder selection procedure. For instance, adhering to a democratic principle could mean that not only a hospital manager but also current and future users should be involved in the development process of digital health. However, criteria have not been proposed to justify the selection of ideal participants.

From a practical point of view, assembling a diverse stakeholder group to design digital technology may require more deliberation in the health care field than in other sectors because the interests of the diverse stakeholders may not be aligned. This may lead to practical challenges for stakeholders in gaining trust and managing multiple stakeholders and time pressure when involving patients and physicians [20-25]. However, design practice manuals do not address how to overcome these additional challenges when using GCD to develop digital health [11,26,27].

When tackling these theoretical and practical issues and involving stakeholders in the GCD process to develop digital health, there is little scientific guidance to help select the best stakeholders. No study has evaluated the performance of different stakeholder groups when using GCD to develop digital health. A meta-review, albeit limited to the development of serious games, has highlighted the need for this research, as the effect of involving some users as stakeholders in PD studies is unclear [28].

### Objective

To provide further scientific guidance on the involvement of stakeholders, we tested the hypothesis that stakeholders with more diverse knowledge and ways of thinking would improve the GCD process. To satisfy this aim, we operationalized the hypothesis through a procedure to assemble distinct stakeholder groups and assess their influence on the GCD process and output. As such, the research question is as follows: *Do stakeholders with diverse knowledge and diverse ways of thinking improve the GCD process for digital health?* The study’s goal is to conduct a preliminary assessment of diverse stakeholder groups assembled through a prescribed procedure in the early stages of a GCD process of a digital health project. This assessment will hopefully provide deeper insights that other researchers and practitioners can consider when deciding the most appropriate stakeholder to involve in their GCD project. With time, this could lead to a validated GCD stakeholder involvement procedure for digital health.

## Methods

### Procedure to Assemble Diverse Stakeholder Groups

**Figure 1:**
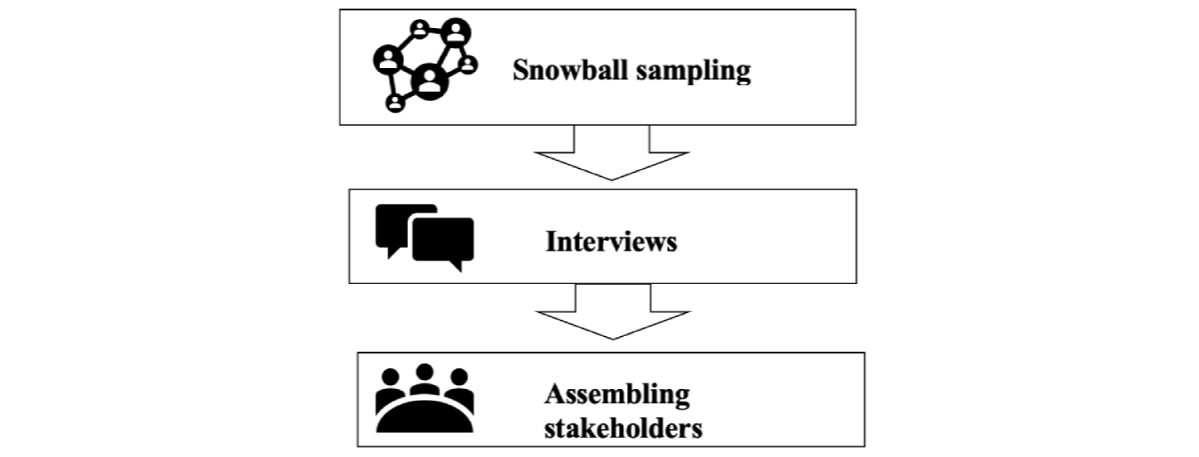
Stakeholder group assembly procedure.

The stakeholder group assembly procedure amounts to the operationalization of the Sanders and Stappers [10] hypothesis that stakeholders with more diverse knowledge and ways of thinking could improve the GCD process. To involve stakeholders who meet these requirements in a GCD process, a procedure containing 3 steps was followed: snowball sampling, interviews, and assemblage of stakeholders (Figure 1).

First, to recruit people, one needs to identify those who are committed to addressing the problem at hand. It can be useful to sample stakeholders through relevant organizations, associations, or events [25,29]. This should help ensure their commitment to solving problems, as these people have directly or indirectly been exposed to the problems and are logically more motivated to develop a solution.

Second, individual interviews can be conducted to qualitatively assess the diversity of knowledge and ways of thinking of the potential members. To operationalize the term “knowledge,” we define 3 types of knowledge (Textbox 1) based on the work of Batens [30-32]. One key form of knowledge that is also defined in GCD research is the deeper-lying tacit knowledge [10], which we measure here as contextual certainties. In addition, there are methodological instructions and relevant statements. Each of these 3 types of knowledge was assessed during an interview on a scale of 0 to 3 (Table 1). Stakeholders with extensive knowledge regarding the relevant statements and contextual certainties will be given the maximum score (3); stakeholders who are uncertain are given a score of 2 and those who seemed to have little knowledge, or did not provide relevant information in the interview, were awarded lower scores (1 and 0, respectively).

To operationalize the other component, “thinking,” we define 4 types of inferences, namely, induction, deduction, abduction-1, and abduction-2 (Textbox 1), as categorized initially by Peirce [33,37,38]. In particular, abduction-2 inferencing is expected to play an important role in the design process [33,38] and is typically attributed to how designers think. Previous experience with these types of inferences can be assessed during an interview by counting the number of times an inference is used (Table 1). Abduction-1 can be scored as the number of methodological instructions formulated as concrete solutions (eg, having an overview of one’s energy capacity after cancer treatment to continue work). Abduction-2 can be scored by looking at the use of generative heuristics as analogies or metaphors.

**Working definitions of knowledge and inference types used for assessment.**

**Knowledge types**

- Contextual certainties
  Knowledge containing a deeper-lying perspective or philosophical principle
- Methodological instructions
  An approach to solve a problem or subproblem such as a procedure for operations, instruments, or tools
- Relevant statements
  Factual knowledge about the problem or the solution

**Inference types**

- Induction
  A sequence of reasoning steps leading to a generalization, whereby several similar utterances are grouped under a new term or name, often in the form of a remark or conclusion following the utterances of others [33]
- Deduction
  A sequence of reasoning steps leading to a conclusion based on several previous utterances [33]
- Abduction-1
  A sequence of reasoning steps leading to the suggestion of a solution in the form of a methodological instruction
- Abduction-2
  A sequence of reasoning steps leading to the suggestion of a solution in the form of a methodological instruction whereby induction, deduction, abduction-1 and generative heuristics can be used, for example, a metaphor [34,35] or analogy [36]

**Table 1:** Criteria used for stakeholder selection.

Assessment aims and criteria			Example interview questions and assessment
**Assess knowledge diversity and depth**			
	Professional background	What is your job?	
	Relevant statements	What, in your view, is the core of the problem about cancer and work? (0-3 score)	
	Contextual certainties	Why is this an important problem? (0-3 score)	
**Assess inference experience**			
	Induction	How did you come upon this problem, through direct or indirect experience? (0-3 score)	
	Deduction	Have you previously tested solutions regarding work and cancer? (0-3 score)	
	Abduction-1 (methodological instructions)	What inspiring solutions arise in your mind to address the work and cancer challenge? (count number of occurrences)	
	Abduction-2	Abduction-1 with generative heuristics as analogies or metaphors (count number of occurrences)	
**Assess communication abilities**			
	Self- assessment	Choice between 3 suggested answers: “OK, but sometimes challenging,” “good,” or “very good”	

In addition, communication skills can be assessed to determine whether potential stakeholders can effectively communicate their ideas to others in a group. For instance, we can assess whether a patient has the appropriate content expert background with various relevant statements that they feel confident to share during a GCD process with other stakeholders by asking the respondent for a self-evaluation.

Third, after conducting the interviews and scoring the responses, a diverse stakeholder group can be assembled based on 3 criteria. One can start by combining people from different professional backgrounds. Next, one can ensure that those stakeholders with the highest knowledge scores are included as they have more knowledge. In other words, if there are 2 stakeholders with the same professional background, the one with the highest score is included. Finally, the diversity of inferencing experience can be assessed. Here, one should ensure that a stakeholder group covers all inference types. Once one is satisfied that the stakeholder group covers all inference types, one can seek out the stakeholders with the greatest inference experience. For instance, if there are 2 stakeholders with abduction-2 experience, the one with the most experience (highest score) can be selected.

### Action Research Approach

To assess the stakeholder group assembly procedure, an action research approach [39] was used to guide the practitioners of a GCD project while adding the stakeholder group assembly procedure to simultaneously gain research insights.

### Hypothesis to Test

The aim was to test how a stakeholder group, assembled using the stakeholder group assembly procedure described in the aforementioned section, would influence the GCD process. We expected that this stakeholder group assembly procedure would produce a group with diverse knowledge and ways and that this would have a positive influence on the GCD process and output. We also expected that, in such a group, the “contextual certainties” knowledge type would be expressed more often by all stakeholders and the “abduction-2” inference type would be more often used specifically by the stakeholders with design expertise than in our less-experienced comparison group.

### Digital Health Project

A digital health development project in which multiple stakeholders could be involved in the GCD process was sought, and we could test the stakeholder assembly procedure to determine if it could make the GCD process more methodologically sound. Given the expertise of the first author (PV) with the problems faced by patients with cancer, a related project was identified and initiated by a Dutch cancer foundation called oPuce (The Foundation). The Foundation aims to create awareness of the stigmatization of cancer and supports initiatives to help people with cancer continue working during and after the illness and promote their return to paid work [40]. The Foundation had planned to start the development of a serious game to help people with cancer address their work-related needs. Although the actual development process had not yet started, The Foundation was interested in using a co-design process to develop the serious game. Because The Foundation had a large network of people who could potentially be involved as stakeholders in the design process to develop the serious game, we chose to add the stakeholder group assembly procedure as a first step in this process and help them with the first GCD activity.

### Ethics Approval

Ethics approval was granted by Erasmus Medical Centre’s Ethics Committee (MEC-2021-0231).

### Assembled Stakeholder Group

#### Overview

The stakeholder group assembly procedure described in the aforementioned section was followed in this study. The research data were solely managed by the first author (PV). The stakeholders received no financial compensation to participate in this study.

Here, we describe how snowball sampling, interviews, and group assembly were carried out. The first author initiated the snowball sampling [41] by approaching people at The Foundation via email and phone to identify stakeholders. At the end of this process, 13 potential stakeholders who had been involved in the initial conversations over the development of a serious game were identified (Table 2).

The 13 potential stakeholders were each assessed through 45-minute interviews, except for the network coordinator with COVID-19. Before the interviews, the participants were informed about the research and asked for informed consent. The web-based audio and video recorded interviews were carried out by PV and facilitated by creative exercises on Miro’s web-based collaborative whiteboard platform (Miro Corp; Multimedia Appendix 1). The creative exercises helped the interviewees gain a visual understanding of their ideas and become accustomed to the web-based creative software they would use during the GCD workshop.

Given that there were multiple stakeholders with similar backgrounds but scored differently in terms of knowledge and inference, the stakeholders could be divided into 2 groups (Tables 3 and 4). A more potent stakeholder group was formed of stakeholders with diverse backgrounds who scored highly on the knowledge and inference criteria. These stakeholders scored high in terms of providing more relevant statements and contextual certainties. This group had experience with all the inference types. A less potent stakeholder group was formed of the remaining stakeholders who still met the desired range of diverse backgrounds but scored less on the knowledge and inference criteria by showing less extensive knowledge and less inferencing experience during the interviews. Notably, none of the stakeholders in this group had experience with abduction-2 inferencing.

The stakeholders in both groups were unaware of this selection procedure, or why they were placed in which group, and the detailed aims of the study.

**Table 2:** Number of potential stakeholders identified through snowball sampling per professional background (N=13).

Background	Stakeholder, n (%)
Game developer and designer	1 (8)
Employer (employing people with cancer)	3 (23)
Employer network	2 (15)
Employed cancer survivor	1 (8)
Occupational physician	1 (8)
Researcher	3 (23)
Network coordinator and patient with a previous history of cancer	1 (8)
IT manager	1 (8)

**Table 3:** Scores of stakeholders in the more potent diverse group.

Background	Score^a^
Game developer and designer	11
Employer (employing people with cancer in company A) and facilitator	11
Employer (employing people with cancer in company B)	9
Employer network	9
Employed cancer survivor	9.5
Occupational physician	10
Researcher	11.5

^a^Average score per stakeholder is 10 (SD 0.95).

**Table 4:** Sores of stakeholders in the less potent diverse group.

Background	Score^a^
Researcher 1	5
Researcher 2	3.5
IT manager	2.5
Employer network	3.5
Employer and facilitator	6
Network coordinator and cancer survivor^b^	10
Ecosystem expert^c^	—^d^

^a^Average score per stakeholder is 5 (SD 2.47).

^b^No formal interview was conducted; information was gathered through informal conversations.

^c^No interview was conducted because this stakeholder only joined as an observer at the start of the generative co-design workshop.

^d^Not available.

#### Data Collection

Data were collected during individual interviews as part of the stakeholder assessment procedure. In addition, data were collected in 2 identical parallel workshops that were part of a larger web-based event organized by The Foundation regarding the working of their organization. Before the workshops, all the stakeholders were given information about the aim of the identical parallel-running workshops, and a link was provided to familiarize themselves with the web-based Miro platform. GCD workshops are social activities in which stakeholders can share knowledge and work with creative exercises toward achieving the purpose of the design project [10,42,43]. Web-based workshops were considered the best option given the COVID-19 pandemic restrictions. The 30-minute web-based GCD workshops were audio and video recorded.

To provide a focus for the assessments, the GCD workshops were slightly artificially divided into 2 phases: the problem phase with the aim to understand the issues to formulate a problem definition and the solution phase to create ideas for a solution. The materials used in the 2 parallel-running GCD workshops were identical and organized specifically to focus on the interactions among stakeholders in both phases. Both groups received 5 identical instructions with a hexagon template delineating both the problem and solution phases, and sticky notes were provided (Multimedia Appendix 1).

In terms of roles, PV similarly facilitated both workshops and switched between them to ensure that the instructions were clear while consciously avoiding steering the content development process. Each stakeholder participated in the respective workshops as a co-designer. In addition, before the workshops, 2 stakeholders were asked if they would take on the double role of a participant and an assistant facilitator. All participants, including the assistant facilitators, were blinded to the hypotheses and aims of the study.

#### Qualitative Analysis

The data from the interviews and workshops were iteratively coded and analyzed using ATLAS.ti (Mac Version 22.1.0; Scientific Software Development GmbH). The influences of the 2 diverse stakeholder groups on the GCD process were assessed in terms of knowledge changes (knowledge output) and how the stakeholders processed the knowledge (the use of inferences). Given this focus, the changes in knowledge were assessed by comparing the knowledge displayed during the initial interviews with that developed during the workshop within both groups.

To compare the 2 workshops, we coded each set of interactions between stakeholders in the problem and solution phases about a certain topic as a sequence in each workshop. In each sequence, we used the deductive and inductive codes described in the following section to be able to compare the knowledge processing of both stakeholder groups in each sequence and phase. We separately compared the sequences of both groups in the problem and solution phases because the knowledge outputs in the problem phase (the problem statement) and solution phase (forms of methodological instructions) were different.

Thematic and inductive codes were used to assess changes in the knowledge from that revealed in the interviews to that in the workshops. The thematic codes were based on the definitions in Textbox 1, using 3 types of knowledge and 4 inference types to assess the knowledge processing and output. Using the same definitions of the assessment criteria during the stakeholder group assembly procedure and workshop analysis ensured that we could compare at the level of knowledge and inference types. The interview data can show that an individual stakeholder mentioned a certain fact (relevant statement type) or a certain approach to finding a solution (methodological instruction type) before joining the GCD process. To evaluate the changes in knowledge possessed by the stakeholders over time, that is, interview through workshop, we used codes such as “repetition from interview” if the knowledge generated in a workshop had already been mentioned by one of its members in their interviews. If the knowledge did change during the workshop, we assessed how it had changed in a particular sequence of interactions between stakeholders.

Thematic inference type codes were used to code group interactions during the GCD workshops. We followed a coding approach similar to that by Cramer-Petersen et al [33], whereby inferences were coded and analyzed in an empirical design setting. As such, utterances that bore similarities to the logical inference forms were coded according to the appropriate inference type (Textbox 1).

To further qualify the knowledge processing and knowledge output identified with the above-described deductive codes, 17 inductive codes (Multimedia Appendix 2) were used to identify stakeholder behaviors (eg, suggest a new idea or a reformulation; Table 5). These were used to understand why certain knowledge or inference types were used in each sequence.

**Figure 2:**
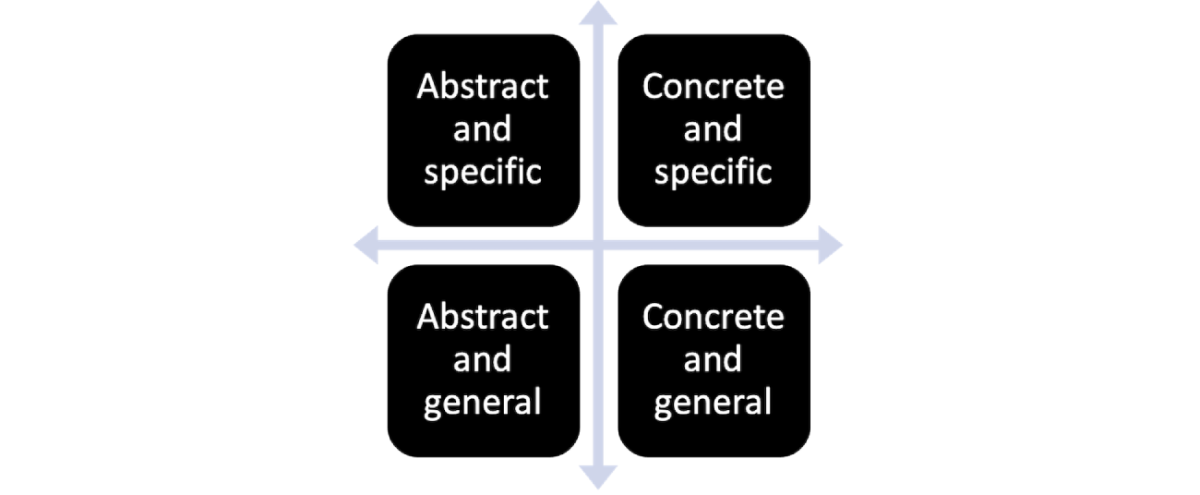
Inductive codes to code the knowledge changes: on x-axis from abstract to concrete and on y-axis from general to specific.

To assess the knowledge output in a sequence during the solution phase, 4 inductive codes were used to code knowledge changes through stakeholder interactions (Figure 2): concrete specific (eg, proposing to use a coach), concrete general (eg, proposing to use artificial intelligence), abstract specific (eg, a virtual angel—a specific object or artifact), and abstract general (eg, an empowering journey—a general image that may contain several specific solutions).

**Table 5:** Examples of inductive code names and definitions to assess changes of knowledge within the workshops (see Multimedia Appendix 2 for complete list).

Code name	Definition
Introduce	Utterance whereby a new idea is proposed
Reformulate	Utterance whereby a previous idea is expressed using different words
Add	Utterance whereby aspects are added to a new idea

## Results

### Main Findings

Our hypothesis on diverse stakeholders was confirmed, as the more potent stakeholder group had a relatively larger influence on the GCD workshop process and output in the problem phase (see *Greater Processing of Relevant Statements Increased Knowledge About the Problem*) and solution phase (see *Greater Use of Abduction-2 Inferencing Improves the Concreteness and Specificity of Solutions*) than the less potent group (Table 6). Regarding the problem phase, in terms of influence on the process, the more potent stakeholders built on each other’s relevant statements, some of which had already been mentioned in the interviews before the workshop. Here, we noticed a dual movement. On the one hand, there was an expansive movement of diverse knowledge as the varied stakeholders shared their knowledge about the problem, and on the other hand, there was a narrowing integrative movement in which the content of ideas changed, and this changed the course of the discussion. In terms of *output*, the more potent group developed a more comprehensive problem definition.

Regarding the solution phase, in terms of influence on the *process*, the more potent group used more abduction-2 inferences, leading to a greater variety of methodological instructions (Table 6). In addition, the more potent diverse stakeholder groups, as in the problem phase, developed each other’s methodological instructions. This made the solutions more concrete and specific. Therefore, in terms of GCD *output* in the solution phase, the more potent stakeholders had a greater influence, as this group produced more precisely described solutions.

The other 2 subhypotheses were not supported. Only once, and only implicitly, contextual certainties were identified in the GCD workshop (Table 6). This was true only among the more potent stakeholder groups. As such, there seems to be no substantial difference between the 2 groups in terms of explicitly sharing more tacit deeper-lying knowledge. Furthermore, although we had expected abduction-2 type inferencing to be applied by stakeholders with a design background, it was not used by the game developer who was the only participant with this background in the more potent diverse stakeholder group. Rather, abduction-2 inferences were made by the nondesigners in this group, which is contrary to our expectations.

**Table 6:** Frequency of codes in interactions in the more potent and less potent stakeholder groups.

Code group and code		Frequency in more potent group				Frequency in less potent group		
		Problem phase		Solution phase	Problem phase			Solution phase
**Inference type**								
	*Induction^a^*	*10*	0		*0*		2	
	*Deduction*	*9*	6		*4*		5	
	Abduction-1	0	2		0		5	
	*Abduction-2*	0	*13*		0		*0*	
**Knowledge type**								
	*Relevant statements*	*14*	4		*10*		6	
	*Methodological instructions*	0	24		0		*8*	
	Contextual certainties	1	0		0		0	

^a^Key differences have been highlighted in italics.

### The Greater Processing of Relevant Statements Increased Knowledge About the Problem

In terms of interactions about the problem, the stakeholders in the more potent group shared a greater diversity of relevant statements (14 vs 10), which were processed using more induction (10 vs 0) and deduction inferences (9 vs 4) than the less potent diverse stakeholder group did (Table 6). Furthermore, the stakeholders in the first group built on each other’s relevant statements, some of which had already been mentioned in the interviews before the workshop. These interactions were related to focusing on the discussion, asking questions, explaining ideas, introducing new ideas, and reformulating old ones, which occurred more frequently in the more potent group.

How stakeholders in the more potent stakeholder group developed each other’s knowledge about the problem is clearly demonstrated in the examples of the more potent group (Table 7). The employer expanded the discussion concerning the self-management of cancer survivors and added that one should consider the resilience of these people and avoid putting them into a victim role. Although he had already mentioned the need for a bespoke resilient solution in the individual interview, this was not in relation to considering the victim role of a patient or in relation to self-management. The employer and facilitator reformulated these points slightly and responded that this comment was related to developing the content of the serious game rather than its implementation. The game developer specified (relevant statement) that these aspects concern the content and didactics behind the content of the serious game. This probably follows from a more abstract principle that the game designers believe in, that “the content of a serious game always has a didactic aim behind it” (contextual certainty). The employed cancer survivor returned to what the employer had mentioned earlier and questioned whether there was a victim role at all. Finally, the employer and facilitator attempted to integrate the different points and reformulate this as a new question.

Thus, in the more potent group, the stakeholders such as employers and a patient shared their views on the problem by asking questions, reformulating points, and trying to draw connections. They shared their different ways of viewing self-management for people with cancer looking forward to returning to work. As a stakeholder, the technological background of the game developer enabled him to quickly point out how this could be accommodated in a serious game through the underlying didactics. This shows how each of the different stakeholders in the GCD process can rapidly interject useful information to define the problem based on the actual needs while conforming to what is technically needed and possible.

The interaction between stakeholders in the less potent group (Table 8) was more a group conversation without people building on each other’s knowledge (relevant statements). This led to less integration of the knowledge that was being shared. Even though they seemed to make a start to focus on the aspect of the problem as “the barriers preventing people with cancer to resume work,” they did not ask each other what that means or attempted to define the barriers. In the more potent stakeholder group, we observed more concentrated attention on the content of the problem, which led to more integration of knowledge about the problem, for example, the concepts of self-management, the victim role, and serious game development were rapidly connected to a problem definition.

**Table 7:** Sequence with codes from more potent diverse stakeholder group (translated into English for reporting purposes).

Stakeholder and sequence of utterances (order of conversation)			Behavior code		Inference-type code		Knowledge-type code		Repetition code
**Employer**									
	1. It feels to me that a user-centered bespoke solution is very general. I mean, doesn’t that apply to any situation?	Focus		Deduction		—^a^		—	
**Employer and facilitator**									
	2. How would you make it more concrete?	Focus and ask		Deduction		—		—	
**Employer**									
	3. For example, coming back to what was said previously, how can we facilitate self-management? How can we avoid creating a victim role?	Introduce		—		—		—	
	Because we want to make something bespoke. For example, how can you contribute to the resilience of the candidates looking for work or those who want to maintain work?	Explain		Deduction		Relevant statement		From interview	
	It’s in line with self-management, but a bit more.	Reformulate		Induction		—		—	
**Employer and facilitator**									
	4. How can you connect that to a serious game? It’s obviously also a general problem.	Ask		Deduction		—		—	
	How do you maintain self-management? How do you prevent the victim role? Then, you are in the development process of the serious game.	Reformulate		Induction		—		—	
**Game developer and designer**									
	5. But more content, the didactics behind it.	Introduce		Induction		Relevant statement and contextual certainty		From interview	
**Employer**									
	6. The content	Reformulate		Induction		—		—	
**Game developer and designer**									
	7. Yes, indeed	Agree		—		—		—	
**Employed cancer survivor**									
	8. If there would be a victim role?	Ask		—		Relevant statement		—	
**Employer and facilitator**									
	9. I am thinking about the last point of (employer) and from (researcher) to keep it concrete and small and still also connect it with the piece on implementation.	Focus		Induce		—		—	
	Then we arrive again at the point of how do we make sure that the serious game offers added value for individual employees with cancer, but then we still remain with a big problem.	Reformulate		Deduce		—		—	

^a^Not available.

Over time, the interactions about the problem in the GCD workshop with the more potent stakeholders showed a dual movement that was not present in the less potent group. On the one hand, there was an expansive movement of diverse knowledge as the stakeholders shared more knowledge about the problem and on the other hand, there was a narrowing integration movement whereby the content of ideas changed, which changed the course of the discussion. For example, initially, there was an expansive diverse knowledge movement as various stakeholders discussed the broad theme of user-centeredness. Then, there was a narrowing integration discussion about the definition of the user, whereby the question was raised as to whether one should focus on the development or implementation aspects. Some aspects were considered together, as it was mentioned that self-management was important for users. Here, the initial ideas changed as this was rephrased to clarify that some aspects are relevant during the development phase of the serious game and others during its implementation. Other elements that were discussed concerned resilience and the victim roles to be considered (Table 8), although these were not integrated into the problem definition. This dual movement may have contributed to the more potent diverse stakeholder group having a more comprehensive problem definition (Textbox 2) than the less potent group. In the problem definition phase, the less potent stakeholder group seemed to have brought together ideas in an expansive movement; however, there was no subsequent integration of or change in the content that formed the problem definition. The more potent group’s more elaborate problem definition seems to have provided a better-founded basis on which to develop solutions.

**Table 8:** Sequence with codes from less potent diverse stakeholder group (translated into English for reporting purposes).

Stakeholder and sequence of utterances (order of conversation)			Behavior code		Inference-type code		Knowledge-type code		Repetition code
**Researcher 1**									
	1. If I am now looking. I am focusing on the serious game. That seems to be the starting point. Then, I think a central problem is that we see that the current ways of people getting back to work are not successful. And we want to improve that. Improve self-management. Well, let’s continue here, I am sure you can add to this.	Introduce		Deduction		Relevant statement		From interview	
**Employer and facilitator**									
	2. Does everyone agree?	Ask		—^a^		—		—	
**Network coordinator and cancer survivor**									
	3. I think also, how can you improve the collaboration? How can you, with each other? Perhaps intercompany or inter-academic? Perhaps, this has nothing to do with…	Introduce and ask		—		—		—	
**Ecosystem expert**									
	4. What I thought is that solution-oriented thinking is more on the outside of the hexagon (exercise template). I think that the word removing barriers to resume work, that is for example a problem related to the content. I don’t know how others are looking at this?	Introduce, reformulate, and ask		—		—		—	
**Researcher 2**									
	5. I agree with that.	Agree		—		—		—	
**Network coordinator and cancer survivor**									
	6. This is about keeping your work?	Ask and reformulate		Deduction		—		—	
**Ecosystem** **expert**									
	7. Keeping your work.	Agree		—		—		—	

^a^Not available.

**Problem definitions.**

**Problem definition of the more potent diverse stakeholder group**

- How do we realize a bespoke approach and self-management during the implementation of the serious game (whilst taking this into account during development of the serious game)?

**Problem definition of the less potent diverse stakeholder group**

- Maintaining work during and after cancer

### Greater Use of Abduction-2 Inferencing Improves the Concreteness and Specificity of Solutions

In the solution phase, the more potent group of diverse stakeholders used more abduction-2 inferences (13 vs 0), which led to a greater variety of methodological instructions (24 vs 8) than those observed in the less potent group (Table 6). In addition, similar to what the stakeholders did in the problem phase, the more potent diverse stakeholder group developed each other’s methodological instructions in the solution phase. This resulted in more concrete and specific solutions. Furthermore, abduction-2 inferencing was used by nondesigners, which was less anticipated because inferencing is typically attributed to designers.

**Figure 3:**
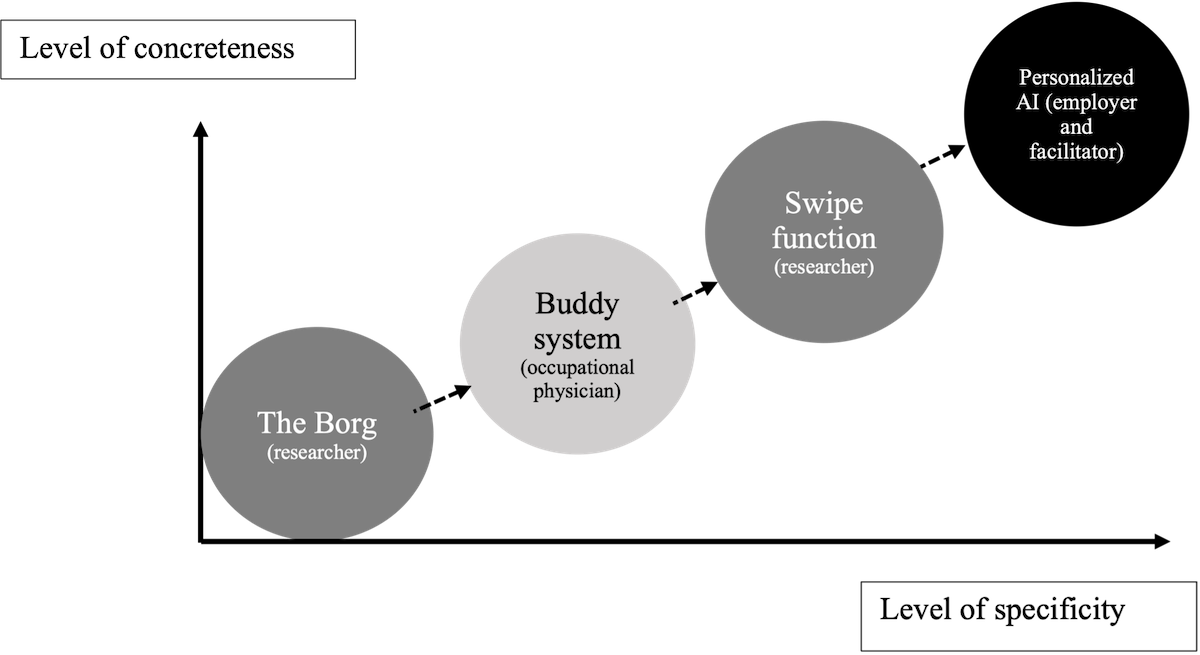
Visualization of iteration of solutions (bubbles) suggested by different stakeholders in terms of specificity and concreteness (different shading for each stakeholder). AI: artificial intelligence.

How stakeholders developed ideas based on each other’s methodological instructions and how this made the solution more concrete and precise are clearly demonstrated in the example of the more potent group (Table 9). The researcher suggested a solution that he explained as being a tool for a social network, using a Star Trek metaphor by referring to The Borg. This is an abstract solution, characterized by a metaphor, yet sufficiently specific, as it is further described as a social network. Next, other suggestions, each using a different metaphor, were used as analogies to highlight different features or aspects of the social network. Thus, the solution became more concrete and specific. The occupational physician suggested a buddy system; the researcher suggested a similar swipe function as in a Tinder app; and the employer and facilitator suggested offering personal suggestions based on an artificial intelligence algorithm. The metaphors that were used seem to have come from popular culture or daily use, which may have made them immediately clear to all stakeholders. As such, the solution-related knowledge of the various stakeholders started on an abstract-specific level and moved toward a more concrete and specific level (Figure 3). Overall, the more potent diverse stakeholder group had a strong influence on the quality of the knowledge output regarding the solution.

The interaction in the less potent group was more on the level of sharing relevant statements about a solution, for example, improving the skills of people with cancer (Table 10). They did not discuss in more detail how skills training could be implemented with, for instance, visual images (abduction-2). Therefore, the solutions did not change from abstract to concrete; instead, they remained relatively the same at a concrete level.

**Table 9:** Example sequence utterances from the more potent diverse stakeholder group in the generative co-design workshop with codes (translated into English for reporting purposes).

Stakeholder and sequence of utterances (order of conversation)			Behavior code		Inference-type code		Knowledge-type code		Repetition code
**Researcher**									
	1. You are not as an individual… because in such a game you are addressed as an individual, so how do we keep the social element and your environment? As an image I have The Borg^a^, that’s from Star Trek, and you are being assimilated in a very large network of other individuals.	Introduce		Abduction-2		Methodological instruction		From interview	
**Game developer and designer**									
	2. I didn’t know you were a Trekkie.	Joke		—^b^		—		—	
**Researcher**									
	3. Wait until you see my costume, ha-ha.	Laugh		—		—		—	
**Occupational physician**									
	4. I am thinking about a sort of buddy system^c^, rather than peers with similar experience, use buddy’s to play together.	Introduce		Abduction-2		Methodological instruction		—	
**Researcher**									
	5. Yes, and maybe we can therefore also connect that with a Tinder app^d^, because which buddy would you like?	Introduce		Abduction-2		Methodological instruction		—	
**Occupational physician**									
	6. Ha-ha.	Laugh		—		—		—	
**Employer and facilitator**									
	7. And, there, the artificial intelligence rises to the surface again? So that you can see on the basis of your use of the game with who you have the best connection^e^?	Introduce		Deduction and abduction-2		Methodological instruction		—	
**Occupational physician**									
	8. Exactly.	Agree		—		—		—	
**Employer and facilitator**									
	9. That you are not only swiping, but also get a suggestion, like Hi, this person could fit with you.	Explain		—		—		—	

^a^First visual image.

^b^Not available.

^c^Second visual image.

^d^Third visual image.

^e^Fourth visual image.

**Table 10:** Example sequence utterances from the less potent diverse stakeholder group in the generative co-design workshop with codes (translated into English for reporting purposes).

Stakeholder and sequence of utterances (order of conversation)			Behavior code		Inference-type code		Knowledge-type code		Repetition code
**Employer network**									
	1. I am still thinking about an approach including skills, how that would enable people. I put it left under (in Miro), I lost it…	Introduce		Abduction-1		Methodological instruction and relevant statement		From interview	
**Network coordinator and cancer survivor**									
	2. No, but skills are really important. Here, you have to do something completely different, and you are looking at work differently.	Agree and add		—^a^		Relevant statement		—	
**Ecosystem expert**									
	3. But I think that next to the work environment also, if you assume that that was the work environment where you were, the other one could then call a different work environment. Then those skills arise again, because you can perhaps get the possibilities to develop yourself differently.	Add		Deduction		—		—	
**Employer network**									
	4. Yes, and when one conquers cancer, for example you have certain perseverance, that you are resilient. And when you focus on that, your employer can you help you realise this.	Add		Conclude		Relevant statement		—	

^a^Not available.

## Discussion

### Principal Findings

This study aimed to answer the following research question: *Do stakeholders with diverse knowledge and diverse ways of thinking improve the GCD process for digital health?* As a first step in attempting to answer this research question, we assessed how a diverse stakeholder group, put together using the proposed stakeholder group assembly procedure, would influence the GCD process. We also established a second stakeholder group consisting of individuals who scored less well in the preliminary interviews held to assess the required competencies.

Our preliminary findings confirm Sanders and Stappers’ main hypothesis that a group of stakeholders with diverse knowledge and ways of thinking has a positive influence on GCD. The more potent of the 2 diverse stakeholder groups had a relatively larger influence on the GCD workshop process and output. The stakeholders in the more potent group built more on each other’s knowledge, which led to a more comprehensive problem definition and more precisely described solutions. In the problem phase, the stakeholders in the more potent group shared a greater diversity of relevant statements (14 vs 10), which were processed using more induction (10 vs 0) and deduction inferences (9 vs 4) than the ones in the less potent diverse stakeholder group. Furthermore, the stakeholders in the first group built on each other’s relevant statements, some of which had already been mentioned in the interviews before the workshop. This resulted through a dual movement toward a more comprehensive problem definition. In the solution phase, the more potent group of diverse stakeholders used more abduction-2 inferences (13 vs 0), which led to a greater variety of methodological instructions (24 vs 8) than those observed in the less potent group. In addition, similar to what the stakeholders did in the problem phase, the more potent diverse stakeholder groups developed each other’s methodological instructions in the solution phase. This resulted in solutions that were developed from a more abstract and general level toward a more concrete and specific level.

The other 2 subhypotheses were not supported. First, there was no substantial difference between the 2 groups in terms of explicitly sharing deeper-lying knowledge (contextual certainties). One contextual certainty was used implicitly in the more potent group. Second, abduction-2 inferences were used 13 times by nondesigners in the more potent group but not by the game designer in the more potent group. This result was contrary to our expectations.

Using a person’s professional background as the sole criterion for group member selection as, for example, done by Trischler et al [44], may not deliver the full potential of a GCD session. Rather, it is the combination of stakeholders with diverse and complementary knowledge in terms of 3 knowledge types (relevant statements, methodological instructions, and contextual certainties) and the most diverse and complementary inference experience in terms of 4 complementary inference skills (deduction, induction, abduction-1, and abduction-2) that enhances the GCD process and its output. Moreover, abduction-2 inferencing did not occur spontaneously in our study in the less potent diverse stakeholder group. Therefore, the involvement of at least one stakeholder with abduction-2 experience (not limited to professional designers) could be critical when using GCD in hierarchical hospital settings [25], with stakeholders who are not naturally involved in creative activities.

Furthermore, the speed brought about by the dual movement of divergence and convergence [45] in the problem phase could be due to the diversity of knowledge and thinking among the stakeholders, as each one has the potential to convergence or diverge. Here, each has knowledge that others lack and cannot think in ways that others can. In the problem phase, the example provided was about an idea that was rapidly considered from a patient experience and from the employer and technical development perspectives. This led to reformulations and the raising of new questions, which steered the process in a new direction. This could be viewed as a change of frame, or perspective, brought about through the interaction of different stakeholders. Although there is extensive literature on the framing process [46-49], the interactions of diverse stakeholders in the framing process have not yet been explicitly described. The example we provided in the solution phase suggests that framing involving diverse stakeholders can be viewed as a knowledge process that looks for a solution from different knowledge contexts that provide different perspectives when looking at a possible solution. During this process, we observed an implicit negotiation process, which has been mentioned by other researchers [47,50], in the sense that the stakeholders’ responses to the proposed solutions varied. On some occasions, stakeholders laughed, which may signify acceptance of a solution. This was surprising and unexpected given that it did not relate to their own knowledge context. As such, a stakeholder group with diverse knowledge and ways of thinking may be the most effective when it can reframe ideas rapidly.

The framing process may be accelerated when stakeholders share more contextual certainties. However, we observed only 1 event in the problem phase that demonstrated how a contextual certainty can rapidly bring a new perspective to a discussion; in this case, a didactic perspective that is essential when developing serious games [51,52]. This emphasizes the need to share deeper-lying knowledge in the GCD process [10] and the need to explicate how they are used by different stakeholders in design theory more broadly [53]. The limited expression of contextual certainties in our study may be due to the lack of priming exercises [8] ahead of our workshops, coupled with the time pressure and workload of participants. This may have suppressed the participants’ awareness of deeper-lying ideas. This suggests that there may be a minimum critical time before people can share such deeper-lying knowledge that our workshops failed to exceed.

### Implications

Finally, we reflect on our stakeholder group assembly procedure in light of the normative values present in the GCD that originate in the PD field [10]. In PD, broadly defined values are upheld such as democracy, equalized power relations, mutual learning, and situation-based actions [16,19]. Given the lack of theoretical consensus, there are no solid normative grounds on which to judge our stakeholder selection procedure. For instance, the democratic principle might imply that one should involve people who are affected by the design decisions made or the end product [19]. In addition, it is emphasized that power relations should be equalized by giving voice to those who may be invisible or weaker [16]. In terms of digital health, this could imply that patients and informal caregivers should be involved. As it is often difficult to get involved in a health care setting [21], we considered the use of a snowball sampling method. This is potentially more inclusive and faster than a widely advertised recruitment strategy that may not attract susceptible groups. As such, in the protocol, we tried to cast a wide net of possible participants through snowball sampling to include people and other vulnerable populations. However, to participate in and contribute to the GCD process, individuals should be able to bring new or complementary knowledge and inferencing experience to the stakeholder group. On the basis that they lacked these assets, we did not include cancer survivors in the more potent diverse stakeholder group, even though they were in a susceptible position. Furthermore, it is argued that democracy requires educated and engaged people acting in their own interests and in the interest of the common good [54,55]. Kensing and Greenbaum [55] state that, when necessary, this should involve educating people in terms of the required technical jargon and engaging them in the process, an aspect related to the principle of mutual learning [16,19,55]. In this respect, Kleinsmann argues that in collaborative activities, there should be minimal shared understanding [56]. In our protocol, we tried to ensure this by looking for people with a basic interest in the topic through snowball sampling and then using self-assessment to evaluate group communication abilities. In this sense, we believe that the stakeholder group assembly procedure that we used can serve as an example of how these values can be respected while improving the GCD process and output.

### Limitations

The designed stakeholder group assembly procedure was operationalized in a minimally viable form to meet the aim and scope of this study. Although the assessment process was intended to accurately score the knowledge, inference skills, and communication skills of potential group members, there may be a built-in bias in the questions. Although we attempted to limit this by discussing the formation of the groups within the research team, there may still be some errors in allocating individuals to one of the 2 groups.

Indeed, not all the criteria were sufficiently sensitive to differentiate between the experiences of some stakeholders to ensure robust selection. For instance, all the stakeholders scored similarly on the criteria addressing induction and deduction inference types and communication abilities. This could be due to the snowball sampling that preselected stakeholders who were already part of The Foundation’s network with a certain level of educational training and communication abilities. Even though all the stakeholders showed a similar ability to use induction and deduction inference types in their interviews, the stakeholders in the less potent group used these less often during their workshop, which affected their knowledge output and knowledge processing. It is possible that the stakeholders in this group were less inclined to use these inference types because of a lack of interaction.

The case was selected based on the background of the lead researcher and the fact that it was a project that had momentum, was about to start, and had good potential to involve various stakeholders. However, the selected case also raised concerns, as it took longer than expected to gain approval to start the stakeholder selection procedure from the project manager. One reason for this could be that GCD is often used as an informal design practice rather than as a formal scientific approach with formal stakeholder selection.

We would caution readers against drawing any causal relationships based on our study about the influence of the stakeholder groups on the GCD process. To maintain focus in our analysis, back-and-forth interactions between the problem and solution phases, which might occur when addressing a real issue, were not considered. Furthermore, given the exploratory purpose of this study, various variables were ignored, including content-related facilitation, interpersonal relationships [57], the creative environment [58], mutual learning over time, and the higher-level strategy of the project and host organization [56,59]. Nevertheless, even without these aspects, this study was still able to provide initial insights into the role of stakeholder diversity in GCD. To ensure this, reflection meetings were organized between the lead researcher and coauthors to identify and avoid any potential biases in the study design and interpretation of the results.

### Further Research

We would recommend further exploring how to strike a balance between the time and resources spent on snowball sampling and the number of stakeholder assessment criteria (knowledge, inference experience, and communication abilities) used. One option would be to ignore induction and deduction and focus on abduction-1 and abduction-2 inference experiences. One could also ignore communication abilities if the organization under consideration is a hospital that already requires interdisciplinary collaboration and focus instead on visual communication skills and open-mindedness as an indication of creative thinking. Next, to further assess the influence of the selected stakeholders on the knowledge processing component, the role of metaphors (in abduction-2 inferencing) and contextual certainties could be explored. For instance, one could link the dual-processing theory of reasoning, which involves deeper unconscious knowledge processing based on intuition and experience, and the more conscious deliberated processing with different knowledge and inference types [60]. Finally, the knowledge processing and knowledge output could, over time, be further assessed in the GCD process, in which the expression of contextual certainties is considered alongside stakeholders’ learning processes.

### Conclusions

A procedure to assess the diversity of knowledge, diversity of ways of thinking, and communication skills in assembling a stakeholder group that meets specific criteria may improve the potential of the GCD process and the resulting digital health. We would encourage the validation of our preliminary findings. Ultimately, this will help researchers make methodologically more robust decisions about stakeholder involvement and report them in an appropriate way, which will improve the scientific rigor of GCD science for digital health.

## Appendix

Multimedia Appendix 1 Interview and workshop template used on online Miro platform.

Multimedia Appendix 2 Inductive code list.

## Abbreviations

GCD: generative co-design
PD: participatory design
