# Supplementary figures and images for: Diversity in Stakeholder Groups in Generative Co-design for Digital Health: Assembly Procedure and Preliminary Assessment
Source: JMIR Hum Factors. 2023 Feb 14;10:e38350. doi: 10.2196/38350 (PMC9975926; doi:10.2196/38350)

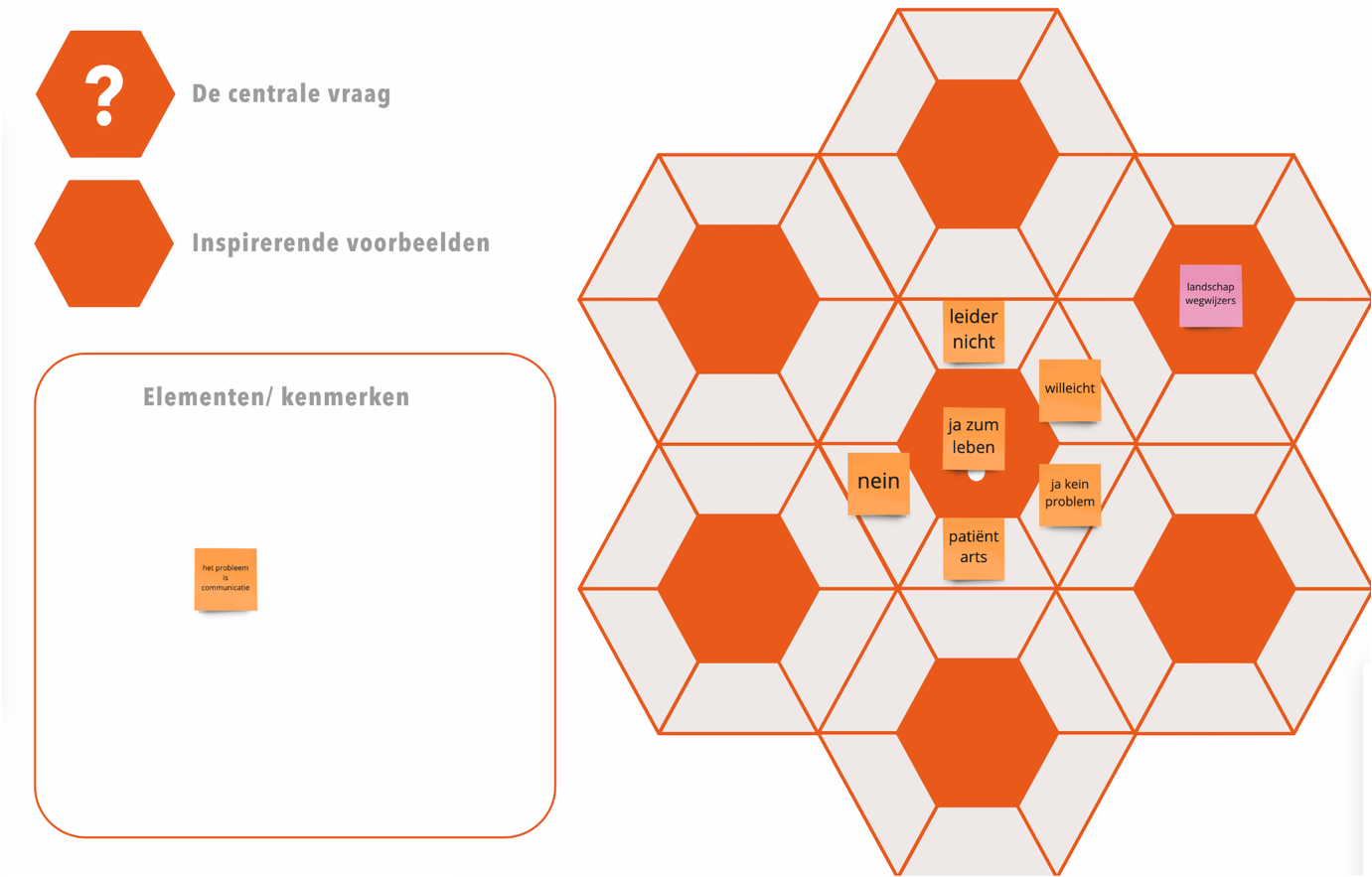

Supplement: Multimedia Appendix 1 [file humanfactors_v10i1e38350_app1.png]
