# Supplementary material for: Diversity in Stakeholder Groups in Generative Co-design for Digital Health: Assembly Procedure and Preliminary Assessment
Source: JMIR Hum Factors. 2023 Feb 14;10:e38350. doi: 10.2196/38350 (PMC9975926; doi:10.2196/38350)
Supplement: Multimedia Appendix 2 [file humanfactors_v10i1e38350_app2.docx]

**Appendix 2: Inductive code list**

| Introduce |
| --- |
| Agree |
| Explain |
| Add |
| Reformulate |
| Ask question |
| Disagree |
| Choose |
| Joke |
| Close discussion |
| Focus |
| Clarify |
| Laugh |
| Understand |
| Make options |
| Misunderstand |
| Repeat |
| Total |
